# Temperature-driven shifts in the epibiotic bacterial community composition of the brown macroalga *Fucus vesiculosus*

**DOI:** 10.1002/mbo3.79

**Published:** 2013-03-13

**Authors:** Stephanie B Stratil, Sven C Neulinger, Henrik Knecht, Anette K Friedrichs, Martin Wahl

**Affiliations:** 1 GEOMAR Helmholtz Centre for Ocean Research KielKiel, Germany; 2 Institute for General Microbiology, Christian-Albrecht-University KielKiel, Germany; 3 ICMB Institute of Clinical Molecular Biology KielKiel, Germany; 4 Department of Internal Medicine, University Hospital Schleswig-HolsteinCampus Kiel, Kiel, Germany

**Keywords:** 16S rRNA gene, 454 pyrosequencing, alga, DGGE, diversity, epibacteria

## Abstract

The thallus surface of the brown macroalga *Fucus vesiculosus* is covered by a specific biofilm community. This biofilm supposedly plays an important role in the interaction between host and environment. So far, we know little about compositional or functional shifts of this epibiotic bacterial community under changing environmental conditions. In this study, the response of the microbiota to different temperatures with respect to cell density and community composition was analyzed by nonculture-based methods (denaturing gradient gel electrophoresis and 454 pyrosequencing of the 16S rRNA gene). Redundancy analysis showed that despite high variability among host individuals temperature accounted for 20% of the variation in the bacterial community composition, whereas cell density did not differ between groups. Across all samples, 4341 bacterial operational taxonomic units (OTUs) at a 97% similarity level were identified. Eight percent of OTUs were significantly correlated with low, medium, and high temperatures. Notably, the family Rhodobacteraceae increased in relative abundance from 20% to 50% with increasing temperature. OTU diversity (evenness and richness) was higher at 15°C than at the lower and higher temperatures. Considering their known and presumed ecological functions for the host, change in the epibacterial community may entail shifts in the performance of the host alga.

## Introduction

The important roles of surface-associated biofilms in transepidermal exchange processes and other interactions between host and environment have recently been described (Wahl et al. 2012). In the past years, studies of the “microbiome,” that is, entire microbial communities in environmental samples, have become possible with the aid of high-throughput sequencing methods (Margulies et al. 2005). These studies have highlighted the diversity, specificity, and dynamics of these communities, for example, in air (Bowers et al. 2011), in water (Gilbert et al. 2012), in soil (Roesch et al. 2007), in sands (Gobet et al. 2012), and associated with hosts. Terrestrial host organisms such as humans (e.g., Costello et al. 2009) and trees (Redford et al. 2010) have been investigated. In the field of biological oceanography, the number of studies which investigate the diversity and ecological role of complex bacterial communities associated with corals (Cárdenas et al. 2012), sponges (Webster et al. 2010; Lee et al. 2011; Jackson et al. 2012; Schmitt et al. 2012), polychaetes (Neave et al. 2012), ascidians (Behrendt et al. 2012), and macroalgae (Bengtsson et al. 2012) are steadily emerging. Marine macroalgae are known to carry diverse bacterial communities (reviewed in Hollants et al. 2013) which chemically interact with their hosts in harmful and beneficial ways (reviewed in Goecke et al. 2010). The hosts provide a rich source of carbons, for example, fucoidan, agar, and alginate that bacteria enzymatically degrade (reviewed in Goecke et al. 2010). The bacterial community on several species of brown, green, and red algae has been previously studied using fingerprinting, cloning, and hybridization methods. These studies have shown that the community is quite species-specific (Lachnit et al. 2009), albeit with a high variability between individuals of one species (Tujula et al. 2010). Furthermore, the community is tissue-specific to some degree (Staufenberger et al. 2008) and significantly differs from the bacterioplankton in the surrounding water column (Bengtsson et al. 2010; Burke et al. 2011a).

The brown macroalga, *Fucus vesiculosus*, a foundation species in the Western Baltic Sea, has been subject of several studies focusing on its interactions with epibacteria. These studies have shown that the epibacterial community, while being species specific (Lachnit et al. 2009), varies seasonally (Lachnit et al. 2011). The intricate biofilm community has been shown to affect further settlement by bacteria and diatoms (K. Laufer and S. Alpert, unpubl. data) and to be involved in the repulsion of barnacle larvae (Nasrolahi et al. 2012), exemplifying possible beneficial functions these epibacteria may have for their host. The host alga itself has also been shown to chemically repel certain bacteria while attracting others (Saha et al. 2011), suggesting that certain members of the bacterial community may be detrimental to the host.

However, information on the detailed structure of the bacterial community on this ecologically important macroalga and how it responds to and possibly mediates environmental shifts is still lacking. Equipped with the tools of high-throughput sequencing, it is possible to take an in-depth look at the structure of microbial communities on marine macroalgae and investigate how they respond to abiotic factors. Temperature is an important abiotic factor in determining the growth of *F. vesiculosus* and possibly its associated bacteria. At elevated temperatures, the alga shows signs of stress (as indicated by weakened antifeeding defenses) (Weinberger et al. 2011) and microfouling pressure becomes more severe (Wahl et al. 2010; M. Fischer, pers. comm.). Considering these findings, we expect an increase of epibacteria on the thallus surface at higher temperatures. Temperature also deserves attention in experiments because at the sea surface it is predicted to increase in the Western Baltic by 0.5–2.5°C (Neumann and Friedland 2011) or even 4°C (BACC Author Team 2008) within the next century, respectively, depending on the model. This will possibly cause stress for the alga species and may restructure its epibacterial community.

In this study, we conducted a laboratory experiment in which *F. vesiculosus* was cultured at different temperatures. We sampled the alga's associated epibacterial community and sequenced the V1–V2 region of the 16S rRNA gene in order to analyze the bacterial community composition. We predicted that temperature will lead to shifts in the bacterial community composition and would increase the overall number of bacterial cells on the thallus.

## Experimental Procedures

### Sampling, experimental design, and setup

Twenty-five individuals of the brown macroalgal species *F. vesiculosus* were collected in the Kiel Bight, Western Baltic (54°27′N, 10°11′E), at the end of October 2009. They were transported to the laboratory in plastic bags within 2 hours of collection. Subsequently, each algal individual was placed in one 25-L aquarium. Algal individuals were incubated for 28 days at five different temperatures: 5, 10, 15, 20, and 25°C (±0.5°C). Replication per temperature level was five, resulting in 25 aquaria with independent bodies of water. In order to assure constant temperature levels, the five tanks of one temperature level were placed in larger water baths which were filled with water that was heated or cooled to the respective temperature. Water was circulated with water pumps (Aqua EL Circulator 350; Aquael GmbH, Erkrath, Germany) in the water baths and in the individual tanks in order to ensure homogenously distributed temperatures. The experiment was run in a constant temperature chamber set at 15°C. We adjusted the higher temperatures (i.e., 20°C and 25°C) with three heating rods (Schego 600; Schemel & Goetz GmbH & Co KG, Offenbach am Main, Germany) per water bath. The lower temperatures (i.e., 5°C and 10°C) were achieved by circulating the water of the water bath through a cooler (Aqua Medic Titanium 1500; Aqua Medic GmbH, Bissendorf, Germany). The temperature levels were maintained constant throughout the experiment by temperature sensors (Aqua Medic T2001HC, Aqua Medic GmbH) that electronically controlled the heating rods; the water coolers had integrated temperature sensors. Water temperatures in the 25 experimental units were furthermore measured at least once a day with a standard digital thermometer to ensure controlled temperature levels, never deviating by more than 0.5°C. The temperature range in the experiment was chosen based on temperatures experienced by *F. vesiculosus* in its natural habitat and based on the prediction for warming of the Baltic Sea due to global change. Temperatures of up to 20°C are experienced by *F. vesiculosus* in its natural habitat during the course of the seasons (Figure S1). Water temperatures of 25°C are rarely reached in the Western Baltic, but can occasionally occur during the summer months in shallow waters (<50 cm) (K. Maczassek, unpubl. data). Light was supplied by metal halide lamps (250 W; 10,000–12,000 K) delivering 100 ± 5 μmol/m^2^/s at the thallus surface and a dark/light regime of 16:8. Water in the tanks had a salinity of 16 and was obtained by mixing 1/3 fjord water and 2/3 artificial seawater (Instant Ocean®, Blacksburg, VA) in order to dilute nutrients and thus reduce diatom growth. Artificial seawater was aged for 48 h before it was distributed to the tanks. Water in the tanks was exchanged weekly. After 0, 7, 14, and 21 days of incubation, the bacteria on the algal surfaces were sampled (always on the day before the water in the tanks was exchanged) for analysis of community composition and after 28 days for quantification of bacterial cells. Two algal fronds per individual were softly rinsed for 10 s with sterile seawater in order to remove loosely attached particles including unattached biofilm components. Then the bacteria on approx. 15 cm^2^ (visual estimation) per branch of young tissue were sampled with a sterile cotton swab (one swab per branch). The swab was placed in a 1.5-mL Eppendorf tube and frozen at −80°C until DNA extraction. Sampled algal fronds were marked with a colored thread in order to prevent resampling.

### DNA extraction

DNA was extracted with the QIAamp DNA Mini Kit (Qiagen GmbH, Hilden, Germany) following the manufacturer's protocol for buccal cotton swabs with an additional incubation step at 95°C for 8 min after the second lysis step of the protocol with buffer AL. DNA was eluted with nuclease-free water and stored at −20°C until further analysis.

### Bacterial community composition via 16S rRNA gene sequence analysis

For a first rough overview of the bacterial community composition at different temperature levels and sampling times, we used the fingerprinting method “Denaturing Gradient Gel Electrophoresis” (DGGE) (Muyzer et al. 1993). We sampled the bacterial community at day 0, 7, 14, and 21 according to the procedure described above. Based on DGGE band patterns, the samples of day 0 (before temperature incubation) did not significantly differ. At day 7, 14, and 21 of temperature incubation, we detected significant differences in the epibacterial community composition by redundancy analysis (RDA, see below) (Figure S2A and B; Table S2; Figure 2). As the shifts were more pronounced after 14 days, indicating that the temperature-driven change was not finalized after 7 days, we chose the samples of day 14 for an in-depth look of the epibacterial community via barcoding and 454 pyrosequencing.

### PCR–DGGE

The V3 region of 16S rRNA gene sequences of bacteria was amplified using the primer pair 341F- GC (5′-[CGC CCG CCG CGC GCG GCG GGC GGG GCG GGG GCA CGG GGG G] CCT ACG GGA GGC AGC AG-3′) and 534R (5′-ATT ACC GCG GCT GCT GG-3′) (Muyzer et al. 1993) (Eurofins MWG Synthesis GmbH, Ebersberg, Germany). Polymerase chain reaction (PCR) amplifications were prepared and subsequent DGGE was performed following the protocol in Nasrolahi et al. (2012) and references therein. Bands (presence or absence) of the DGGE fingerprint were called by the bare eye because we have found that this method is less error prone than band calling by computer software. DGGE band counts were treated as operational taxonomic units (OTUs) in subsequent RDA (see below).

### 454 pyrosequencing

Samples were prepared for pyrosequencing following the procedure described in Rehman et al. (2011). Briefly, fragments of ∼450 base pairs (bp) of the V1–V2 hypervariable region of the 16S rRNA gene were amplified using the primer pair 27F (5′-AGAGTTTGATCCTGGCTCAG-3′) with 454 Life Sciences (Roche, Penzberg, Germany) adapter B (not shown) and 338R (5′-TGCTGCCTCCCGTAGGAGT-3′) with 454 Life Sciences adapter A (not shown). The reverse primer contained a multiplex barcode identifier sequence (10 bp) which allowed identification of individual samples. After PCR amplification in duplicates per sample, the DNA was purified and quantified (for details see Rehman et al. 2011). Equimolar amounts of DNA from the 25 samples were pooled and amplicon libraries were sequenced with a 454 GS-FLX pyrosequencer using the Titanium Sequencing Kit (Roche, Penzberg, Germany) at the Institute of Clinical Molecular Biology (ICMB), Kiel, Germany.

### Denoising of sequence data

With the software PANGEA (Giongo et al. 2010), individual samples were sorted according to their barcode (Perl script command *barcode.pl*) and low-quality reads were removed from the data set using a Phred quality score of 20. Further denoising steps were done with the program mothur (Schloss et al. 2009). Sequences with homopolymers longer than 8 bp, with ambiguous bases, and with errors in the primer and barcode sequences were removed (*trim.seqs*). Barcodes and primers were removed before sequences were aligned (*align.seqs*) with the SILVA rRNA database (Pruesse et al. 2007) and screened for a minimum length of 200 nucleotides (*screen.seqs*). Vertical gaps in aligned sequences were removed (*filter.seqs*). Chimeric sequences were identified and removed using the *chimera.uchime* command. Sequences were binned at the 97% similarity level to form OTUs by average neighbor clustering.

### Taxonomic assignment of OTUs

Reads were classified using the Greengenes 16S rRNA reference database (DeSantis et al. 2006) (*classify.seqs*) with the following parameters: method = Bayesian, ksize = 8, iters = 1000, bootstrap cutoff = 60. Sequences identified as chloroplast sequences (44 reads) were removed from the data set before further analysis as these may have originated from the host algae and/or microalgae and do not represent bacteria. OTUs were linked with the results of the classification using the command (*classify.otu*). For comparison to the Greengenes classification, the 15 OTUs listed in Table 2 were also classified using the RDP (Ribosomal Database Project) Bayesian classifier (Wang et al. 2007).

1352 high-quality sequences per sample were randomly subsampled for calculation of diversity indices and for redundancy analysis.

These sequence data have been submitted to the NCBI Sequence Read Archive under accession No. SRX195663-SRX195687.

### Redundancy analysis

RDA was used to analyze the OTU abundance data along the temperature gradient. RDA is a constrained linear ordination method. As opposed to unconstrained ordination methods (e.g., principal component analysis, PCA), which are used for exploratory analysis, constrained ordination explains the relationship between response variables (e.g., species; in our study: OTUs) and explanatory variables (environmental variables; in our study: temperature). Conceptually, RDA is a multivariate linear regression analysis followed by PCA (Borcard et al. 2011). RDA, which is based on Euclidean distances between samples, has been deemed inappropriate for analysis of community count data with many zeros. This is because the Euclidean distance between samples which do not have any species in common (as indicated by the zeros in data tables) is smaller than between samples with some species in common. However, transformations for use on community count data have been introduced which make the method suitable for RDA analysis (Legendre and Gallagher 2001). In the study at hand, OTU abundances were Hellinger transformed. This transformation downweights highly abundant OTUs while avoiding overweighting of rare OTUs (as opposed to, e.g., the chi-square metric; see formula and discussion in Legendre and Gallagher (2001)). RDA was thus done on Hellinger distances between samples (i.e., Euclidean distance of Hellinger-transformed data). As RDA is a linear method, nonlinear OTU responses to temperature (*T*) may not be accurately represented by the analysis. An elegant solution to this problem is to use a second-degree explanatory variable (in our case, *T*^2^) which models unimodal responses (Borcard et al. 2011). In order to assess the individual contribution of *T* and *T*^2^ to the total variance variation partitioning was done (Borcard et al. 2011). In this way, we were able to detect OTUs linearly correlated with temperature, as well as those showing a unimodal response to temperature. The latter are negatively correlated with the squared temperature term (cf. Borcard et al. 2011). OTUs were divided into three categories according to Pearson's correlation coefficient: positive correlation with *T*, negative correlation with *T*, and negative correlation with *T*^2^. All correlations within the same category with a coefficient of determination (*R*^2^) of at least 0.15 were tested for significance, applying Benjamini–Hochberg correction to account for multiple testing. As an additional criterion to prioritize significant OTUs in the analysis, we took their vector lengths in the RDA correlation biplot: the longer the vector of an OTU, the higher its contribution to the set of constrained axes (i.e., the visualized variance) underlying the plot. Analyses were done with R (R Development Core Team 2012) using the vegan package for multivariate analysis of ecological communities (Oksanen et al. 2012).

### Bacterial diversity

In order to assess to which degree bacterial diversity was captured, Good's coverage was calculated by dividing the number of OTUs that had been observed once by the total number of OTUs in the sample. OTU richness was plotted individually for all samples. Evenness, a measure of the homogeneity in relative abundance of the different OTUs making up the richness, is expressed by the Inverse Simpson Index. For statistical analysis of diversity measures, regressions were done in R. The Akaike information criterion (AIC) was used to test the relative goodness of fit for the regressions on temperature. It showed that the binomial model improved the fit over the linear model for the regression of OTU richness on temperature, whereas this was not the case for the regression of the Inverse Simpson Index on temperature (Table S3).

### Quantification of epibacterial cells

In order to assess the effect of temperature on bacterial cell density, direct cell counts on the surface of the thalli at a magnification of 630× were conducted by epifluorescence microscopy (AxioImager.Z1, with a motorized Z-axis lifting table, Zeiss, Jena, Germany) from samples that had been incubated at the experimental temperatures for 4 weeks. We followed the protocol by Bengtsson et al. (2010), adjusting it to the needs of our algal material. 0.5 cm^2^ of algal tissue (younger parts below the meristematic tips) per sample was stained with 0.2% (v/v) 4,6-diamidino-2-phenylindole (DAPI, Life Technologies GmbH, Darmstadt, Germany) for 10 min (*n* = 3 per temperature level, except for 5°C where *n* = 2). Tissue samples were placed on glass slides, a drop of mounting medium (Roti®-Mount FluorCare DAPI, Roth, Germany) was applied, and cover slips were fixed with small pieces of adhesive poster tack. Due to the uneven microtopography of the thallus surface of *F. vesiculosus*, bacterial cells were present in several focal planes. Therefore, stack images (between 5 and 180 single layers of 0.2 μm distance, depending on the individual tissue) of 7–10 visual fields per sample were taken with a monochromatic camera (AxioCam MRm, Zeiss). Before hand counting the bacteria captured on the images, background noise, mostly due to autofluorescence of the host algae cells, was removed. The convolve filter (settings: [-1 -1 -1 -1 -1\-1 -1 -1 -1 -1\-1 -1 24 -1 -1\-1 -1 -1 -1 -1\-1 -1 -1 -1 -1], max. intensity) in the software ImageJ (Schneider et al. 2012) removed most of the background noise. Denoised stacks were converted into Z-projects resulting in all layers combined in one single image. The image was overlaid with a digital counting grid of 50 μm^2^ grid size (ImageJ plugin “grid”). As the number of cells on one single picture exceeded a reliably countable cell density, cells in 20 randomly predefined and evenly distributed squares (each 50 μm^2^) per picture were counted. The mean number of cells per cm^2^ was calculated.

## Results

In a laboratory setup, we investigated the effect of temperature (5, 10, 15, 20, and 25°C) on the bacterial community composition (*n* = 5 per temperature level) after 14 days of incubation and on cell density (*n* = 3 per temperature level, except for *n* = 2 at 5°C) after 28 days of incubation. Since the time points of sampling differed for logistical reasons, we will not relate the bacterial cell densities to the pyrosequencing data and discuss them separately.

Pyrosequencing yielded 133,343 reads across all 25 samples. After the quality check 26% of reads were discarded; that is, 99,142 sequences with an average read length of 300 bp remained in the data set. A total of 4341 OTUs across all samples grouped at the 97% similarity level were identified. Good's coverage ranged from 78% to 85% (Table S1) indicating that the entire diversity was not captured.

### Effect of temperature on community composition

**Figure 1:**
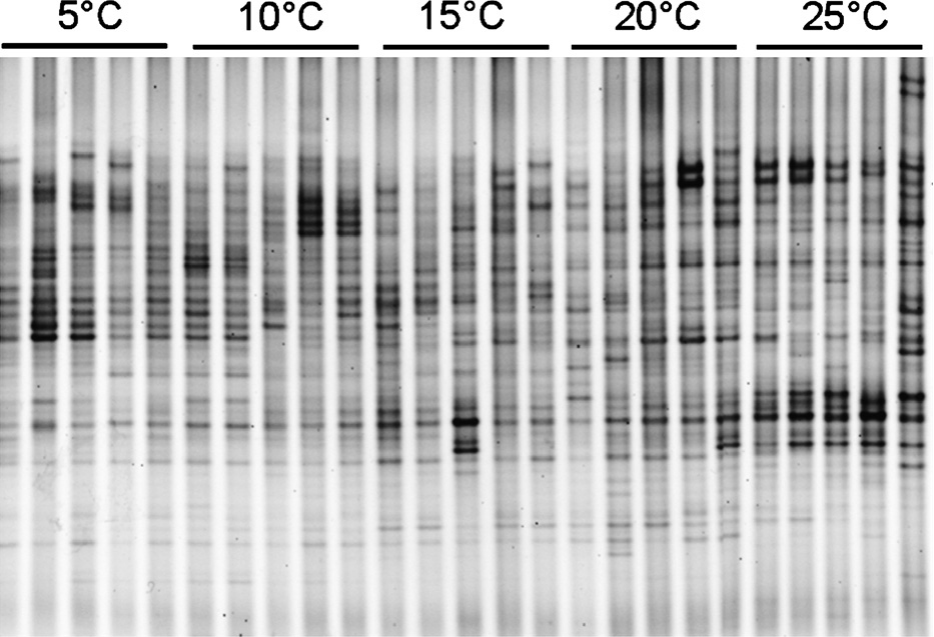
Denaturing gradient gel electrophoresis (DGGE) fingerprint of 16S rRNA gene sequences (V3 region) of epibacteria from *Fucus vesiculosus* cultured at different temperatures for 14 days (5, 10, 15, 20, and 25°C; *n* = 5 per temperature level).

**Figure 2:**
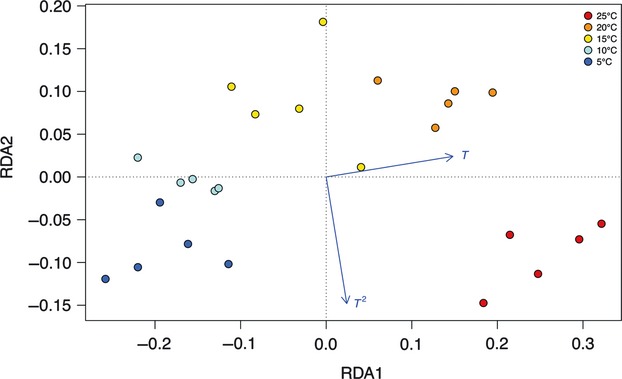
Redundancy analysis of the Hellinger-transformed presence absence data matrix based on bands of the denaturing gradient gel electrophoresis (DGGE) fingerprint of epibacteria from *Fucus vesiculosus* cultured at different temperatures for 14 days (5, 10, 15, 20, and 25°C; *n* = 5 per temperature level). Distance biplot showing relationships between samples (scaling 1) as depicted by the sample points and the temperature gradient vectors (*T* for linear relations and *T*^2^ for unimodal relations).

**Figure 3:**
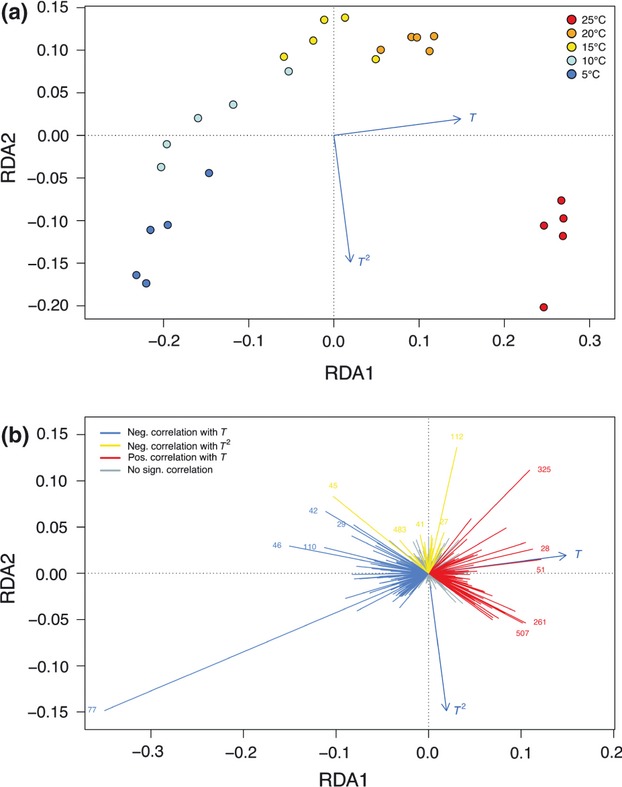
Redundancy analysis of the abundances of different bacterial groups (i.e., operational taxonomic units [OTUs]) associated with the macroalga *Fucus vesiculosus* incubated at different temperatures for 14 days (5, 10, 15, 20, and 25°C; *n* = 5 per temperature level). Data was obtained by 454 pyrosequencing. (a) Distance biplot showing relationships between samples (scaling 1) as depicted by the sample points and the temperature gradient vectors (*T* for linear relations and *T*^2^ for unimodal relations). (b) Correlation biplot (scaling 2) showing lines for OTUs with highest species scores. OTU vectors pointing in the direction of the temperature vectors (*T*) correlate positively with the variable. OTU vectors pointing in the opposite direction of the *T* vector correlate negatively with the variable. OTUs correlating with *T*^2^ point in the opposite direction of the respective *T*^2^ vector (due to the negative quadratic term in the unimodal model). OTUs with a larger influence on the model are indicated by longer vectors, whereas OTUs with vector tips near the origin of the plot are of lesser importance.

**Figure 4:**
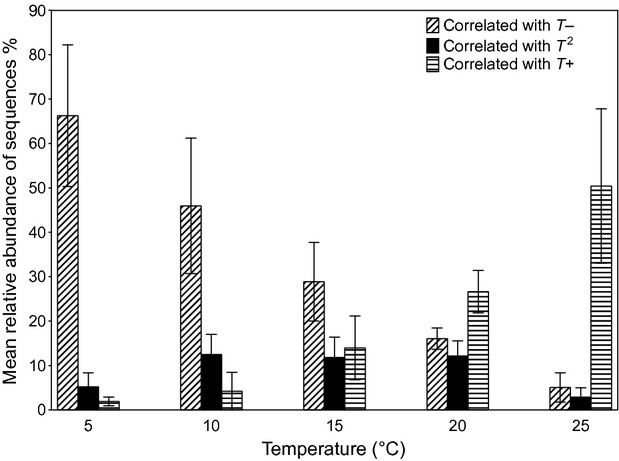
Cumulative relative abundances of operational taxonomic units (OTUs) that were negatively correlated with temperature (correlated with *T*−), positively correlated with temperature (correlated with *T*+), and negatively correlated with the squared temperature term (correlated with *T*^2^), that is, showing an unimodal response to temperature. Bars represent the mean of five replicates per temperature level and error bars represent ±95% CI.

A significant effect of temperature on the bacterial community composition was found by RDA of the DGGE data based on the band pattern in the gel (Fig. 1) and the 454 pyrosequencing reads. Samples separated along the temperature gradient (Figs 2, 3A) as indicated by the distances between sample points which represent 2-D approximations of their Hellinger distances. The explanatory variable temperature (modeled by *T* and *T*^2^) accounted for approx. 25% of variation in the DGGE data and 20% in the pyrosequencing data. Adding *T*^2^ improved the fit of the model of the pyrosequencing data by explaining 4% of the variation (RDA Axis 2, Table 1). Some OTUs were significantly correlated with temperature as indicated by the angles between the lines of OTUs and the explanatory variable (Fig. 3B). One hundred and eighty-four OTUs increased in abundance with temperature, 151 OTUs decreased in abundance with increasing temperature, and 30 OTUs showed a unimodal response to temperature indicating that they were most abundant at the intermediate temperature (15°C). The 15 OTUs with the strongest correlations, as indicated by vector length, are listed in Table 2 and shown in Fig. 3B. Of these, three were nearly absent (<5 reads) at the lower temperature levels (OTU # 325, 261, 507) and two were nearly absent at the high temperature levels (OTU # 77, 46) (Table S4). Correlated OTUs made up 8% of the total number of OTUs and ∼61% of reads (averaged for the five samples of each temperature level). The cumulative relative abundances of OTUs that were negatively correlated with temperature decreased from 65% to 5% with increasing temperature. The ones that were positively correlated with temperature increased in abundance from 2% to 50% with increasing temperature. OTUs that were negatively correlated with *T*^2^ (i.e., had the highest abundance at the intermediate temperature) increased from 5% to 12% and then decreased again to <5% (Fig. 4).

**Table 1:** Amount of variation explained by temperature (*T*) and the squared temperature term (*T*^2^) in the redundancy analysis of the Hellinger-transformed presence absence data matrix based on bands of the denaturing gradient gel electrophoresis (DGGE) fingerprint and of the pyrosequencing data from epibacteria associated with *Fucus vesiculosus* cultured at different temperatures for 14 days

	% Variation explained	

Data set	*T*	*T*^2^
Pyrosequencing	16	4
DGGE	22	3

**Table 2:** The top 15 operational taxonomic units (OTUs) that were negatively correlated with *T* (abundant at low temperatures), negatively correlated with *T*^2^ (abundant at medium temperatures), and positively correlated with *T* (abundant at high temperatures)

	Classification		Correlation with temperature			
	
# OTU	Greengenes	Ribosomal database project (sab score)	Neg.	Neg.*T*^2^	Pos.	Vector length
77	Unclass. *α*-Proteobacteria	*Hellea balneolensis* (0.8)	x			0.54
46	Unclass. Proteobacteria	*Loktanella rosea* (0.94)	x			0.21
42	Unclass. Rhodobacteraceae	*Loktanella maricola* (0.95)	x			0.18
110	Unclass. *α*-Proteobacteria	Unclass. *α*-Proteobacteria	x			0.16
29	Erythrobacter longus	*Erythrobacter longus* (0.92)	x			0.14
112	Unclass. Myxococcales	Unclass. Myxococcales		x		0.20
45	Unclass. *γ*-Proteobacteria	*Granulosicoccus antarcticus* (0.96)		x		0.19
483	Unclass. Rhodobacteraceae	*Loktanella koreensis* (0.91)		x		0.07
27	Unclass. Loktanella	*Loktanella rosea* (0.94)		x		0.07
41	Unclass. *δ*-Proteobacteria	Unclass. Proteobacteria		x		0.06
325	Unclass. *α*-Proteobacteria	Unclass. *α*-Proteobacteria			x	0.22
51	Unclass. Rhodobacteraceae	Unclass. Rhodobacteraceae			x	0.17
261	Unclass. Rhodobacteraceae	Unclass. Rhodobacteraceae			x	0.17
28	Unclass. Rhodobacteraceae	Unclass. Rhodobacteraceae			x	0.16
507	Unclass. Rhodobacteraceae	Unclass. Rhodobacteraceae			x	0.16

### Diversity of the epibacterial communities

**Figure 5:**
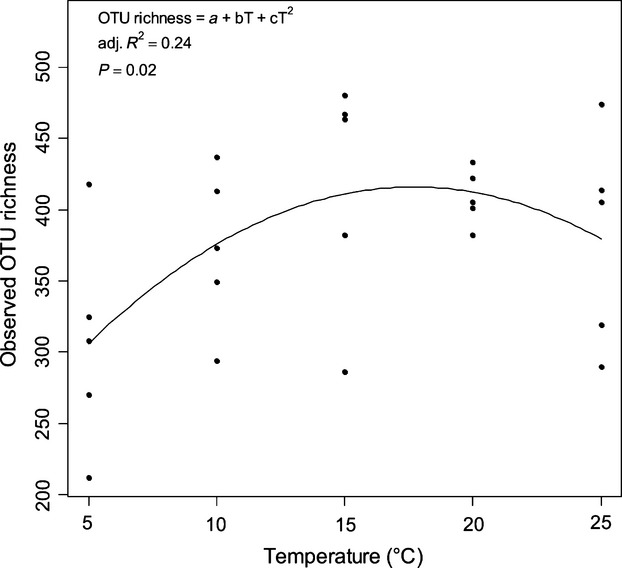
Operational taxonomic unit (OTU) richness at different temperatures (*n* = 5 per temperature level).

**Figure 6:**
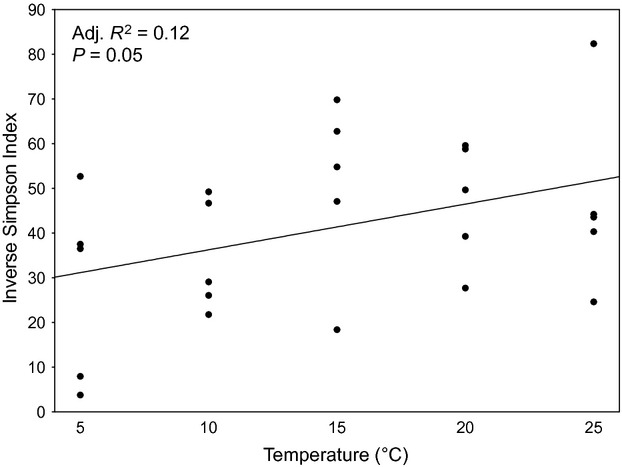
Evenness of operational taxonomic units (OTUs) at different temperatures: inverse simpson index (*n* = 5 per temperature level).

OTU richness was significantly higher at 15°C but variation in OTU richness between replicates of one temperature level was high resulting in a relatively low explanatory quality of the model (Fig. 5). The Inverse Simpson Index is an evenness index which takes OTU abundance into account; the higher the value the more balanced are the abundances of OTUs in the community. The lowest values were found in two of the 5°C samples indicting that they were dominated by a few taxa. Overall, the trend of higher evenness in the 15°C samples is also seen here, however, variation between the samples is high and the single highest evenness is found in a 25°C sample (Fig. 6).

### Taxonomic classification

**Figure 7:**
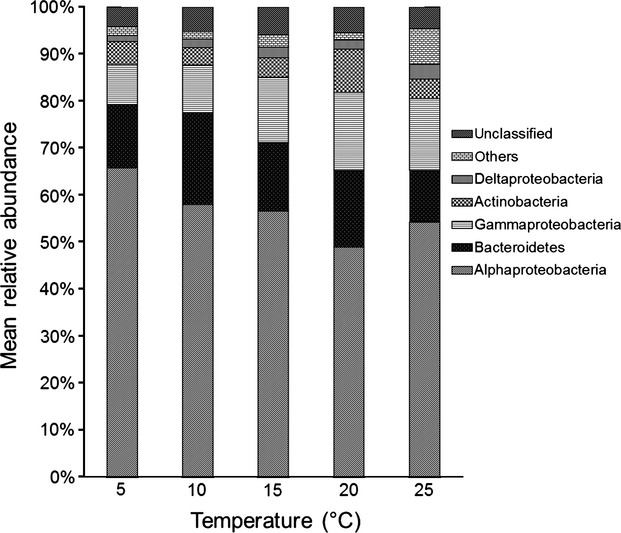
The top five phyla: relative abundances of operational taxonomic units (OTUs) (97% similarity). The phylum Proteobacteria is subclassified into Alpha-, Gamma-, and Delta proteobacteria. The rest of the phyla are compiled in “others.”

Ninety-two percent of the OTUs were classified into 21 phyla while ∼8% of the OTUs could not be classified. At the phylum level, most of the OTUs were classified as Proteobacteria (∼68%) and Bacteroidetes (∼18%) across all samples. The other 19 phyla together made up ∼6% of the reads. The class Alphaproteobacteria was the most abundant at all temperatures (65% at 5°C to 53% at 25°C, Fig. 7). At the family level, Rhodobacteraceae were the most abundant in all samples (at least 20%) and they more than doubled in abundance from 5°C to 25°C (Figure S3). The 15 OTUs that had the most influence on the compositional shifts driven by temperature were classified in the Greengenes and RDP databases. In the latter, classification at lower taxonomic levels (i.e., genera or species) could be obtained in some cases (Table 2).

### Effect of temperature on cell density

**Figure 8:**
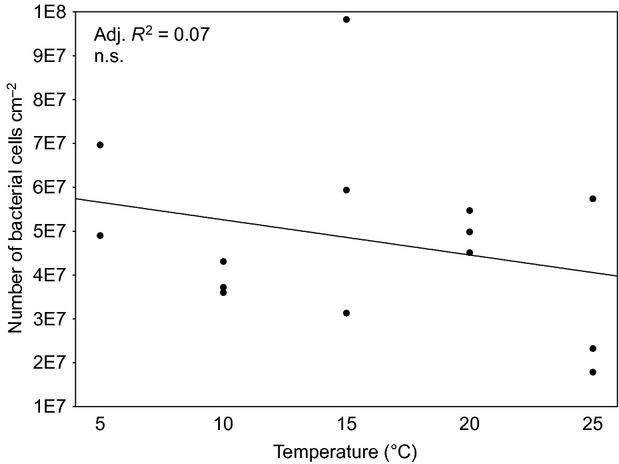
Number of 4,6-diamidino-2-phenylindole (DAPI) stained bacterial cells on the surface of *Fucus vesiculosus* after 28 days of temperature treatment. *N* = 3 except for *n* = 2 at 5°C.

The number of bacterial cells ranged from 1.5 × 10^7^ to 1 × 10^8^ cells per cm^2^ of algal surface with an average cell density of 5 × 10^7^ per cm^2^. Variability between replicates of one temperature level was high (Fig. 8).

## Discussion

In order to assess the effect of temperature on the bacterial community composition associated with the thallus surface of the brown macroalga, *F. vesiculosus,* we chose the fingerprinting method DGGE as a way to give a first overview of potential community shifts. Even though DGGE has limitations (such as comigration of different phylotypes into one band (Sekiguchi et al. 2001), the method was suitable as a first and conservative exploration of shifts that were due to the temperature treatment (as shown by redundancy analysis (RDA) explaining 25% of the variation). The more sophisticated method of 454 pyrosequencing confirmed the results based on DGGE by explaining a similar amount of variation (20%) in the data set by RDA. Despite the fact that different regions of the 16S rRNA gene were amplified (V3 and V1/V2, respectively) the results of both methods were strikingly similar. Based on these results, we find the combination of inexpensive DGGE fingerprinting for exploratory purposes followed by pyrosequencing for a more detailed look at the bacterial community a suitable method for identifying the effects of environmental factors on the bacterial community composition.

Temperature accounted for 20% of the compositional differences in epibiotic bacterial communities on the host groups exposed to different temperatures. This leaves a large proportion of variation to be explained by factors not controlled in our experiment. As all other abiotic factors such as light and salinity were kept identical among treatment groups (as far as possible), biotic factors such as host alga physiology, as well as diverse interactions between the bacteria, host, and other members of the biofilm community may have covaried among treatment groups and somewhat blurred the direct temperature effect. It is likely that random processes also contributed to the variability in the community composition observed in our study. In studies on epibacterial communities on a green macroalga species, the authors observed high taxonomic diversity and variability of epibacteria (Tujula et al. 2010; Burke et al. 2011a) with a set of core functional genes (Burke et al. 2011b) on different algal individuals of the same species. The lottery hypothesis (Sale 1976) serves as an explanation for these observations, asserting that any bacterial taxon from within a functional guild can occupy space on the host alga if it gets there first (Burke et al. 2011a,b). Thus, it combines random and deterministic processes in explaining epibacterial community assembly.

At the higher taxonomic levels, Proteobacteria (namely Alpha-, Gamma-, Delta proteobacteria), Bacteroidetes, and Actinobacteria were the most abundant taxa found on *F. vesiculosus* in all temperature groups. These are very diverse groups and they are found in many different environments and in/on host organisms (Lachnit et al. 2011; Cárdenas et al. 2012; Jackson et al. 2012; Schmitt et al. 2012). Taxa that were positively correlated with temperature could not be classified any further than to family level and mostly belonged to the Rhodobacteraceae. While temperature had an influence on community composition, it did not significantly affect the density of epibiotic bacterial cells on the surface of *F. vesiculosus*. The observed high interindividual variability corresponds to findings from the brown alga species *Laminaria hyperborea* (Bengtsson et al. 2010). If *F. vesiculosus* regulates the absolute number of bacteria on its surface, the alga's capacity for control was not impaired by the temperature treatment. We had expected a direct and indirect influence of temperature on epibacterial density. Direct, because settlement rates, metabolism, and division rates of planktonic and epibiotic bacteria should increase with warming; indirect, because the host alga appears to perform best at intermediate temperature (Wahl et al. 2010; Weinberger et al. 2011). The indirect temperature effect might alter the microhabitat at the thallus surface as well as the host's capacity to chemically control microfouling. Interestingly, this was not the case. Either temperature does not affect these traits of host and epibacteria, or direct and indirect effects neutralize each other. These aspects await further investigations.

Barcoded pyrosequencing allows production of 16S rRNA gene sequence libraries much larger than those produced with Sanger sequencing (Margulies et al. 2005) for many samples in one run (Hamady et al. 2008). Despite the sophistication of this method there has been some concern that it overestimates diversity (Kunin et al. 2010). In order to minimize this effect, we used a stringent quality check and then binned the OTUs at a 97% similarity level (rather than at 98 or 99%). This is commonly done in studies (Lee et al. 2011; Schmitt et al. 2012) and has been suggested as an acceptable threshold by Kunin et al. (2010). Even though the quality check was rigorous, 4341 OTUs across all samples, that is, on 25 individuals of one alga species, were identified. This number is high when compared with the number of OTUs (also binned at a 97% similarity level) found on leaves of 56 terrestrial tree species (5476 OTUs), on 32 species of marine sponges (2567 OTUs) (Redford et al. 2010; Schmitt et al. 2012), and also on a kelp species (1108 OTUs) (Bengtsson et al. 2012). A direct comparison of OTU richness to most other studies is not easy, due to differences in the quality check of the sequence data, in sequence length, or in the different variable regions of the targeted 16S rRNA gene. Differences in these experimental factors can lead to differing diversity estimates (Engelbrektson et al. 2010). Despite this limitation, bacterial richness seems to be exceptionally high on *F. vesiculosus* (keeping in mind that the entire diversity may not even have been sampled as indicated by Good's coverage, which did not reach 100%). The high OTU richness in our study is due to the large number of rare reads indicating that the “rare biosphere” (Sogin et al. 2006) makes up a large part of the epibacterial community on *F. vesiculosus*. It has been shown that members of the rare biosphere can become very abundant after disturbance (Sjoestedt et al. 2012). In our study, only 8% of the 4341 OTUs were significantly correlated with temperature, indicating that a large part of the bacterial community was unaffected by temperature, as far as their abundances are concerned. However, these 8% of OTUs made up 60% of reads on average. In a study by Bengtsson et al. (2012) on the diversity of kelp-associated bacteria, the community was also dominated by a few abundant OTUs. As evenness was low, the kelp blades were suggested to be “low-diversity habitats.” While we observed the same pattern (dominance of a few OTUs), we conclude that algae surfaces can also be viewed as “conditional high-diversity habitats” as OTUs that are rare under certain conditions may become very abundant when conditions change, thus possibly contributing to interactions between host and microbiota. In our study, there were three OTUs (# 325, 261, 507; see Table S4) that were rare (defined as <5 reads) or absent at lower temperatures and more abundant in the higher temperature treatments. Inversely, two OTUs were abundant at lower temperatures and were rare at higher temperatures (# 77, 46; see Table S4). When a bacterial group practically disappears from the algal surface a potential ecological function may no longer be maintained – unless the vanishing species is replaced by a functionally similar one. If the replacing strain is functionally different, new interactions may arise. Despite substantial variation between replicates, the observation of maximum richness and evenness at 15°C is interesting. In this context, it is noteworthy that Nasrolahi et al. (2012) found that microbial assemblages (dominated by bacteria) harvested from the surface of *F. vesiculosus* had the highest repellant effect on barnacle larvae when the host algae were cultured at 15°C (as compared to 5 and 20°C). Epibacterial diversity was not assessed in the Nasrolahi study. Although our results unequivocally show that the epibiotic biofilms change in composition and diversity in response to warming, we do not know as yet how this reorganization affects ecological functions of the biofilm. Taxonomically different bacteria may form guilds whose members are functionally equivalent as has been shown before for the epibacterial community on a green macroalga (Burke et al. 2011b). The risk, however, exists that they do not.

High summer temperatures, that are expected to increase in the Western Baltic in the course of climate change (BACC Author Team 2008; Neumann and Friedland 2011), will likely cause stress for the macroalga whose conspecifics in the North Sea grow best at 15°C (Lüning 1985) and whose chemical antifeeding defenses may be jeopardized (Weinberger et al. 2011). With increasing temperatures an increasing risk of disease associated with benthic organisms has been observed, for example, in corals (Bally and Garrabou 2007) and in the red alga *Delisea pulchra* (Case et al. 2011). In the latter study, a bacterium from the Roseobacter clade was identified as the pathogen that causes bleaching in the red alga *D. pulchra* (Case et al. 2011; Fernandes et al. 2011). The infections by this pathogen are observed more during the summer time and were shown to be more severe at a high temperature. The pathogen belongs to the family Rhodobacteraceae, a group that correlated positively with temperature in our experiment (Table 2). While this result is noteworthy, not all bacteria from the Roseobacter clade or from other members of the Rhodobacteraceae family are pathogens, so the risk of disease for *F. vesiculosus* at elevated temperatures cannot be discussed further at present. Numbers of other potentially pathogenic bacteria, such as Vibrionaceae and Alteromonadaceae, have also been correlated with increasing temperatures in corals and in seawater (reviewed in Wahl et al. 2012). In our study, members of the Vibrionaceae and Alteromonadaceae were present on the alga surface, albeit in extremely low numbers. Only one of over 4000 OTUs, an unclassified Alteromonadaceae, increased in abundance with increasing temperature but still was present only in very low number.

In this study, we have shown that temperature has a distinct effect on the community structure of epibacteria on the macroalga *F. vesiculosus*, but that there are still more factors that account for the observed variability between host individuals. Some of these factors that are deemed most important for the ecology of *F. vesiculosus* and its epibacterial community apart from temperature (such as salinity and irradiance) will be addressed in a subsequent study. The temporal dynamics of temperature-driven shifts will have to be investigated in long-term studies. Changing temperatures, as predicted for the future, may alter the bacterial communities and possibly their function, unless these communities are resilient and return to their original composition or are functionally redundant. Our study adds evidence that host interindividual variability in bacterial community composition is a widespread pattern, observed in humans, terrestrial plants, and several marine organisms. If the dominant members of the community that are affected by temperature also are the active and functionally important members of the biofilm community, a shift due to temperature may alter the interactions between bacteria and their macroalgal host and therefore possibly their interactions with macrofoulers and grazers. In the future, metagenomic and metatranscriptomic studies could shed light on these questions.

## Supporting Information

**Figure S1.** Mean (circle in the center) water temperature at approx. 1.5 m water depth at the sampling site of *Fucus vesiculosus* during the course of 1 year (May 2009 to May 2010) with standard deviation (thick lines) and minimum and maximum values (thin lines).

**Figure S2.** Redundancy Analysis of the Hellinger-transformed presence absence data matrix based on bands of the DGGE fingerprint of epibacteria from *Fucus vesiculosus* cultured at different temperatures for 7 days (A) and 21 days (B) (5, 10, 15, 20, and 25°C; *n* = 5 per temperature level, except for *n* = 4 at 25°C). Distance biplot showing relationships between samples (scaling 1) as depicted by the sample points and the temperature gradient vectors (*T* for linear relations and *T*^2^ for unimodal relations).

**Figure S3.** Relative abundances of OTUs at the family level. For clarity, only the ten most abundant families are individually depicted in the bars; the rest of the classified families are compiled in “others.”

**Table S1**. Good's coverage in %. Mean of five replicates of each temperature level with standard deviation.

**Table S2.** Amount of variation explained by temperature (*T*) and the squared temperature term (*T*^2^) in the redundancy analysis of the Hellinger-transformed presence absence data matrix based on bands of the DGGE fingerprint of epibacteria from *Fucus vesiculosus* cultured at different temperatures for 7 days and 21 days (see also Figure S2A and B).

**Table S3.** Statistical analysis of community diversity: Comparison of goodness of fit of regressions (AIC, Akaike Information Criterion) and statistical output.

**Table S4.** The top 15 OTUs: Number of cumulative reads of the respective OTU across all replicates (*n* = 5) of one temperature level (the number of total reads per temperature level was 6740).

## References

[b1] BACC Author Team (2008). Assessment of climate change for the Baltic Sea Basin.

[b2] Bally M, Garrabou J (2007). Thermodependent bacterial pathogens and mass mortalities in temperate benthic communities: a new case of emerging disease linked to climate change. Glob. Change Biol.

[b3] Behrendt L, Larkum AWD, Trampe E, Norman A, Sørensen SJ, Kuhl M (2012). Microbial diversity of biofilm communities in microniches associated with the didemnid ascidian *Lissoclinum patella*. ISME J.

[b4] Bengtsson MM, Sjøtun K, Øvreås L (2010). Seasonal dynamics of bacterial biofilms on the kelp *Laminaria hyperborea*. Aquat. Microb. Ecol.

[b5] Bengtsson MM, Sjøtun K, Lanzen A, Øvreås L (2012). Bacterial diversity in relation to secondary production and succession on surfaces of the kelp *Laminaria hyperborea*. ISME J.

[b6] Borcard D, Gillet F, Legendre P (2011). Numerical ecology with R.

[b7] Bowers RM, Sullivan AP, Costello EK, Collett JL, Knight R, Fierer N (2011). Sources of Bacteria in outdoor air across cities in the Midwestern United States. Appl. Environ. Microbiol.

[b8] Burke C, Thomas T, Lewis M, Steinberg P, Kjelleberg S (2011a). Composition, uniqueness and variability of the epiphytic bacterial community of the green alga *Ulva australis*. ISME J.

[b9] Burke C, Steinberg P, Rusch D, Kjelleberg S, Thomas T (2011b). Bacterial community assembly based on functional genes rather than species. Proc. Natl Acad. Sci. USA.

[b10] Cárdenas A, Rodriguez-R LM, Pizarro V, Cadavid LF, Arévalo-Ferro C (2012). Shifts in bacterial communities of two caribbean reef-building coral species affected by white plague disease. ISME J.

[b11] Case RJ, Longford SR, Campbell AH, Low A, Tujula N, Steinberg PD (2011). Temperature induced bacterial virulence and bleaching disease in a chemically defended marine macroalga. Environ. Microbiol.

[b12] Costello EK, Lauber CL, Hamady M, Fierer N, Gordon JI, Knight R (2009). Bacterial community variation in human body habitats across space and time. Science.

[b13] DeSantis TZ, Hugenholtz P, Larsen N, Rojas M, Brodie EL, Keller K (2006). Greengenes, a chimera-checked 16S rRNA gene database and workbench compatible with ARB. Appl. Environ. Microbiol.

[b14] Engelbrektson A, Kunin V, Wrighton KC, Zvenigorodsky N, Chen F, Ochman H (2010). Experimental factors affecting PCR-based estimates of microbial species richness and evenness. ISME J.

[b15] Fernandes N, Case RJ, Longford SR, Seyedsayamdost MR, Steinberg PD, Kjelleberg S (2011). Genomes and virulence factors of novel bacterial pathogens causing bleaching disease in the marine red alga *Delisea pulchra*. PLoS ONE.

[b16] Gilbert JA, Steele JA, Caporaso JG, Steinbrueck L, Reeder J, Temperton B (2012). Defining seasonal marine microbial community dynamics. ISME J.

[b17] Giongo A, Crabb DB, Davis-Richardson AG, Chauliac D, Mobberley JM, Gano KA (2010). PANGEA: pipeline for analysis of next generation amplicons. ISME J.

[b18] Gobet A, Boeer SI, Huse SM, Quince JEE, van Beusekom C, Sogin ML (2012). Diversity and dynamics of rare and of resident bacterial populations in coastal sands. ISME J.

[b19] Goecke F, Labes A, Wiese J, Imhoff JF (2010). Chemical interactions between marine macroalgae and bacteria. Mar. Ecol. Prog. Ser.

[b20] Hamady M, Walker JJ, Harris JK, Gold NJ, Knight R (2008). Error-correcting barcoded primers for pyrosequencing hundreds of samples in multiplex. Nat. Methods.

[b21] Hollants J, Leliaert F, Willems O, De Clerck A (2013). What we can learn from sushi: a review on seaweed–bacterial associations. FEMS Microbiol. Ecol.

[b22] Jackson SA, Kennedy J, Morrissey JP, O'Gara F, Dobson ADW (2012). Pyrosequencing reveals diverse and distinct sponge-specific microbial communities in sponges from a single geographical location in Irish Waters. Microb. Ecol.

[b23] Kunin V, Engelbrektson A, Ochman H, Hugenholtz P (2010). Wrinkles in the rare biosphere: pyrosequencing errors can lead to artificial inflation of diversity estimates. Environ. Microbiol.

[b24] Lachnit T, Blümel M, Imhoff JF, Wahl M (2009). Specific epibacterial communities on macroalgae: phylogeny matters more than habitat. Aquat. Biol.

[b25] Lachnit T, Meske D, Wahl M, Harder T, Schmitz R (2011). Epibacterial community patterns on marine macroalgae are host-specific but temporally variable. Environ. Microbiol.

[b26] Lee OO, Wang Y, Yang JK, Lafi FF, Al-Suwailem A, Qian PY (2011). Pyrosequencing reveals highly diverse and species-specific microbial communities in sponges from the Red Sea. ISME J.

[b27] Legendre P, Gallagher ED (2001). Ecologically meaningful transformations for ordination of species data. Oecologia.

[b28] Lüning K (1985). Meeresbotzranik: Verbreitung, Ökophysiologie und Nutzung der marinen Makroalgen.

[b29] Margulies M, Egholm M, Altman WE, Attiya S, Bader JS, Bemben LA (2005). Genome sequencing in microfabricated high-density picolitre reactors. Nature.

[b30] Muyzer G, Uitterlinden EC, De Waal AG (1993). Profiling of complex microbial populations by denaturing gradient gel electrophoresis analysis of polymerase chain reaction-amplified genes coding for 16S rRNA. Appl. Environ. Microbiol.

[b31] Nasrolahi A, Stratil SB, Jacob KJ, Wahl M (2012). A protective coat of microbes on macroalgae: inhibitory effects of bacterial biofilms and epibiotic microbial assemblages on barnacle attachment. FEMS Microbiol. Ecol.

[b32] Neave MJ, Streten-Joyce C, Glasby CJ, McGuinness KA, Parry DL, Gibb KS (2012). The bacterial community associated with the marine polychaete *Ophelina sp*. 1 (Annelida: Opheliidae) is altered by copper and zinc contamination in sediments. Microb. Ecol.

[b33] Neumann T, Friedland R, Schernewski G, Hofstede J, Neumann T (2011). Climate Change Impacts on the Baltic Sea. Global change and baltic coastal zones.

[b34] Oksanen J, Blanchet G, Kindt R, Legendre P, Minchin P, O'Hara R (2012). http://CRAN.R-project.org/package=vegan.

[b35] Pruesse E, Quast C, Knittel K, Fuchs BM, Ludwig W, Peplies J (2007). SILVA: a comprehensive online resource for quality checked and aligned ribosomal RNA sequence data compatible with ARB. Nucleic Acids Res.

[b36] R Development Core Team (2012). R: A language and environment for statistical computing. http://www.R-project.org/.

[b37] Redford AJ, Bowers RM, Knight R, Linhart Y, Fierer N (2010). The ecology of the phyllosphere: geographic and phylogenetic variability in the distribution of bacteria on tree leaves. Environ. Microbiol.

[b38] Rehman A, Sina C, Gavrilova O, Hasler R, Ott S, Baines JF (2011). Nod2 is essential for temporal development of intestinal microbial communities. Gut.

[b39] Roesch LF, Fulthorpe RR, Riva A, Casella G, Hadwin AKM, Kent AD (2007). Pyrosequencing enumerates and contrasts soil microbial diversity. ISME J.

[b40] Saha M, Rempt M, Grosser K, Pohnert G, Weinberger F (2011). Surface-associated fucoxanthin mediates settlement of bacterial epiphytes on the rockweed *Fucus vesiculosus*. Biofouling.

[b41] Sale PF (1976). Reef fish lottery. Nat. Hist.

[b42] Schloss PD, Westcott SL, Ryabin T, Hall JR, Hartmann M, Hollister EB (2009). Introducing mothur: open-source, platform-independent, community-supported software for describing and comparing microbial communities. Appl. Environ. Microbiol.

[b43] Schmitt S, Tsai P, Bell J, Fromont J, Ilan M, Lindquist N (2012). Assessing the complex sponge microbiota: core, variable and species-specific bacterial communities in marine sponges. ISME J.

[b44] Schneider CA, Rasband WS, Eliceiri KW (2012). NIH Image to ImageJ: 25 years of image analysis. Nat. Methods.

[b45] Sekiguchi H, Tomioka N, Nakahara T, Uchiyama H (2001). A single band does not always represent single bacterial strains in denaturing gradient gel electrophoresis analysis. Biotechnol. Lett.

[b46] Sjoestedt J, Koch-Schmidt P, Pontarp M, Canback B, Tunlid A, Lundberg P (2012). Recruitment of members from the rare biosphere of marine bacterioplankton communities after an environmental disturbance. Appl. Environ. Microbiol.

[b47] Sogin ML, Morrison HG, Huber JA, Mark Welch D, Huse SM, Neal PR (2006). Microbial diversity in the deep sea and the underexplored “rare biosphere”. Proc. Natl Acad. Sci. USA.

[b48] Staufenberger T, Thiel V, Wiese J, Imhoff JF (2008). Phylogenetic analysis of bacteria associated with *Laminaria saccharina*. FEMS Microbiol. Ecol.

[b49] Tujula NA, Crocetti GR, Burke C, Thomas T, Holmstrom C, Kjelleberg S (2010). Variability and abundance of the epiphytic bacterial community associated with a green marine Ulvacean alga. ISME J.

[b50] Wahl M, Shahnaz L, Dobretsov S, Saha M, Symanowski F, David K (2010). Ecology of antifouling resistance in the bladder wrack *Fucus vesiculosus*: patterns of microfouling and antimicrobial protection. Mar. Ecol. Prog. Ser.

[b51] Wahl M, Goecke F, Labes A, Dobretsov S, Weinberger F (2012). The second skin: ecological role of epibiotic biofilms on marine organisms. Front. Microbiol.

[b52] Wang Q, Garrity GM, Tiedje JM, Cole JR (2007). Naive Bayesian classifier for rapid assignment of rRNA sequences into the new bacterial taxonomy. Appl. Environ. Microbiol.

[b53] Webster NS, Taylor MW, Behnam F, Luecker S, Rattei T, Whalan S (2010). Deep sequencing reveals exceptional diversity and modes of transmission for bacterial sponge symbionts. Environ. Microbiol.

[b54] Weinberger F, Rohde S, Oschmann Y, Shahnaz L, Dobretsov S, Wahl M (2011). Effects of limitation stress and of disruptive stress on induced antigrazing defense in the bladder wrack *Fucus vesiculosus*. Mar. Ecol. Prog. Ser.

